# Assessment of Varicella Surveillance and Outbreak Control Practices — United States, 2012

**Published:** 2014-09-12

**Authors:** Adriana S. Lopez, Meredith Lichtenstein, D. Scott Schmid, Stephanie Bialek

**Affiliations:** 1 Division of Viral Diseases, National Center for Immunization and Respiratory Diseases, CDC; 2 Council of State and Territorial Epidemiologists

Case-based varicella (chickenpox) surveillance is important for monitoring the impact of the varicella vaccination program. In 2002, the Council of State and Territorial Epidemiologists (CSTE) recommended that all states move toward case-based varicella surveillance by 2005; in 2003, varicella was made nationally notifiable (Table 1) (1). To ease the transition to case-based reporting, CSTE and CDC recommended starting with sentinel site or outbreak surveillance and then moving to statewide case-based surveillance when feasible. To gauge progress in varicella surveillance, in 2012 CDC and CSTE developed a survey for assessing varicella surveillance practices, which CSTE administered to all states and the District of Columbia (DC). As of 2012, varicella was reportable in 44 (86.3%) of the 51 jurisdictions surveyed, of which 37 (84.1%) conduct statewide case-based surveillance. Of the 38 jurisdictions conducting statewide or sentinel site varicella case-based surveillance, more than 84% reported collecting information on age, sex, and race/ethnicity (all 97.4%), vaccination status (94.7%), outbreak association (86.8%), and disease severity (84.2%). Nineteen (43.2%) of the 44 jurisdictions where reporting was mandated transmitted varicella-specific data to CDC using Health Level 7 (HL7) messaging. Currently, HL7 messaging is the only mechanism available for states to send varicella-specific data to CDC. Although public health agencies have made much progress to strengthen varicella surveillance throughout the United States (2), strategies are needed to facilitate transmission of varicella-specific data to CDC from all jurisdictions, using HL7 messaging, and to increase the number of jurisdictions collecting the varicella-specific data necessary to monitor varicella epidemiology and the impact of the vaccination program nationally.

The CDC and CSTE assessment addressed several important aspects of varicella surveillance, including 1) whether varicella is a reportable condition in the state; 2) type and breadth of surveillance conducted (e.g., statewide case-based, outbreak only, case-based in sentinel sites, or aggregate); 3) varicella-specific variables collected (e.g., vaccination status, disease severity [number of lesions, hospitalizations, complications, and deaths], laboratory testing and results, and clinical and epidemiologic data); 4) types of reporting sites; 5) whether varicella surveillance data are sent to CDC via HL7 messaging; 6) whether laboratory testing for varicella is performed in the state; 7) varicella vaccination requirements for school entry; and 8) outbreak control policies. The assessment was pilot-tested in five states and the final version distributed via e-mail in September 2012 to all state epidemiologists.

All 51 jurisdictions (50 states and DC) completed the assessment. Forty-four (86.3%) indicated that varicella is reportable in their jurisdiction. Among these 44 jurisdictions, varicella cases are reported by schools (42 jurisdictions, 95.4%), hospitals (40, 90.9%), and health care providers (37, 84.1%). A total of 38 jurisdictions (86.4%) conducted case-based surveillance, either statewide or at regional sentinel sites, and 20 (45.4%) conducted surveillance only for varicella outbreaks or cases associated with outbreaks (Table 2).

Among reporting variables, more than 84% of the 38 jurisdictions conducting statewide or sentinel site varicella case-based surveillance reported collecting information on age, sex, and race/ethnicity (all 97.4%), vaccination status (94.7%), outbreak association (86.8%), and disease severity (84.2%) (Table 3). Outcome data, including hospitalizations and deaths, were collected by 35 (92.1%) and 34 (89.5%) jurisdictions, respectively (Table 3). Collection of clinical information ranged from 28 jurisdictions (57.9%) for treatment (i.e., medication or type) to 36 jurisdictions for rash onset date and laboratory testing (both 94.7%) (Table 3).

Varicella-specific data were transmitted to CDC via HL7 messaging by 19 (43.2%) of the 44 jurisdictions. Of the 22 (50%) jurisdictions that did not send data via HL7 messaging in 2012, 14 (63.6%) had not transitioned to HL7 standards. Seven (31.8%) either had other methods for sending data, were planning to transition to HL7 messaging, or were not collecting case-based data, and one (4.5%) had no plans to transition to HL7. Barriers hindering transition to HL7 messaging included competing priorities and lack of staff and funds. Three jurisdictions reported not knowing whether they were sending HL7 messages to CDC.

Of the 51 jurisdictions, 49 (96.1%) reported providing public notification of varicella outbreaks and recommending vaccination as an outbreak control strategy. Other reported control strategies included exclusion from the outbreak setting of 1) patients (34 jurisdictions, 66.7%), 2) persons without evidence of immunity who refuse vaccination (33, 64.7%), 3) persons not up-to-date on vaccinations who refused vaccination (18, 35.5%), and 4) immunocompromised persons or pregnant women without evidence of immunity (27, 52.9%). Overall, 31 (60.8%) of the 51 jurisdictions reported having state guidelines for varicella outbreak control.

A total of 41 (80.4%) of the 51 jurisdictions reported having laboratory capability for varicella testing, and 17 (33.3%) routinely provided such testing. In 36 (70.6%) jurisdictions, most testing was conducted as part of outbreak investigation and control. Testing by polymerase chain reaction (PCR) and culture, the most commonly reported types of varicella tests, were available in 26 jurisdictions (51.8%), and the varicella-zoster virus immunoglobulin G test was available in 24 jurisdictions (47.1%).

In 2012, 13 (25.5%) of the 51 jurisdictions reported requiring only 1 dose of varicella vaccine for school entry, 20 (39.2%) reported having a 2-dose school entry requirement, and 17 (33.3%) reported having both 1-dose and 2-dose school entry requirements depending on the grade level. One (2%) jurisdiction reported having no varicella vaccination requirement for school entry.

## Discussion

Because a large number of varicella cases occurred in the United States at the beginning of the varicella vaccination program (estimated at 4 million cases each year, which approximated the size of the U.S. birth cohort) and varicella was not included as a nationally notifiable condition, nationwide reporting of every varicella case was not feasible at that time (3). In the absence of robust national varicella surveillance, beginning in 1995, data from active surveillance sites were used to monitor impact of the 1-dose varicella vaccination program, and later, the 2-dose program that was recommended in 2006 and implemented in 2007 (2,4). As varicella vaccination coverage increased nationwide (5), and the number of varicella cases decreased, CSTE recommended that states move to case-based varicella reporting by 2005 (6). The findings in this report update an assessment conducted in 2004 and document a 63.0% increase in the number of jurisdictions that mandated varicella reporting, from 27 jurisdictions in 2004 to 44 in 2012 (1,3). Since 2004, varicella surveillance has been greatly strengthened, with 38 (86.4%) of the jurisdictions that mandate varicella reporting now conducting statewide or sentinel site case-based reporting. In nearly all jurisdictions (95.4%) varicella cases are reported by schools. However, hospitals and health care providers also are important sources of reporting, particularly for cases in adults and infants. As varicella incidence continues to decline and vaccination coverage increases, monitoring disease severity, outcomes, and epidemiology among all age groups, including those not targeted for vaccination, remains important.

What is already known on this topic?

National varicella surveillance data are important for monitoring trends in varicella epidemiology. In 2002, the Council of State and Territorial Epidemiologists recommended that varicella be added to the list of nationally notifiable conditions by 2003 and that all states move to case-based reporting for varicella by 2005.

What is added by this report?

As of 2012, varicella has been a reportable condition in 44 of 51 jurisdictions; 38 jurisdictions were conducting statewide or sentinel site case-based surveillance for varicella. However, only 19 jurisdictions had the capability to send varicella-specific data to CDC through Health Level 7 electronic messaging. Among the 51 jurisdictions, 80.4% had the laboratory capacity to test specimens for varicella, and 60.8% of jurisdictions had guidelines for outbreak control. Additionally, all jurisdictions except one had either a 1-dose or 2-dose varicella vaccine school entry requirement.

What are the implications for public health practice?

Continued work by jurisdictions to collect and improve completeness of reporting of all relevant clinical and epidemiologic data, disease severity and outcomes, and vaccination status, along with full implementation of Health Level 7 systems to allow jurisdictions to send their varicella-specific data to CDC will be useful for continued monitoring of the varicella vaccination program and guiding future varicella vaccination policy.

As the varicella vaccination program matures and more cases occur among vaccinated persons, laboratory confirmation is increasingly necessary. Diagnosis of breakthrough disease (i.e., varicella in vaccinated persons) is challenging because disease is often mild and might resemble other rash illnesses or insect bites. PCR testing of lesion specimens has been shown to be the most sensitive and specific for diagnosing varicella (7,8). With the majority of jurisdictions now able to perform laboratory testing for varicella, laboratory confirmation of varicella cases is increasingly feasible and will improve the accuracy of surveillance data. A real-time PCR method was deployed to all state laboratories in 2002 for ruling out smallpox in suspected cases of bioterrorism. The permissible uses for this assay have now been expanded to include confirmation of varicella outbreaks and verification of suspected cases of severe varicella. Vaccine-preventable disease reference centers also are available in the state public health laboratories of Wisconsin, New York, Minnesota, and California for varicella-zoster virus PCR testing, discriminating between vaccine and wild-type strains, and varicella-zoster virus genotyping.

Because varicella disease in vaccinated persons is usually mild, with fewer lesions than in unvaccinated persons, confirming and investigating varicella outbreaks in the 2-dose vaccine era can be challenging and resource intensive (9). Approximately 60% of jurisdictions have developed guidelines for varicella outbreak control.* Although 96.1% of jurisdictions reported recommending vaccination as part of their outbreak control strategies, only 64.7% reported excluding persons without evidence of immunity who refuse vaccination. Such exclusion is an important strategy for controlling outbreaks and for protecting those at risk for severe disease who have not been vaccinated.

Currently, most jurisdictions conducting case-based surveillance collect varicella-specific information; however, fewer than half are able to send those data via HL7 messaging to CDC. HL7 messaging is the only mechanism available to states for sending the varicella-specific data they collect to CDC.† Jurisdictions report that resource limitations remain an important barrier to implementing HL7 messaging.

Considerable progress has been made in national varicella surveillance, and national data are now used to monitor trends in varicella incidence. More complete reporting of all relevant clinical and epidemiologic data, disease severity and outcomes, and vaccination status, along with full implementation of HL7 messaging is needed so that CDC can receive the varicella data collected by jurisdictions and use those data to fully monitor the impact of the varicella vaccination program and guide future varicella vaccination policy.

## Figures and Tables

**TABLE 1:** History of national varicella surveillance and related events — United States, 1972–2007*

Year	Surveillance milestone
1972	Varicella becomes a nationally notifiable disease.
1981	Varicella is removed from the nationally notifiable diseases list.†
1991	The Council of State and Territorial Epidemiologists (CSTE) recommends that states develop or maintain sources of varicella surveillance data (e.g., active surveillance in health maintenance organizations or cities/counties/schools, sentinel reporting systems, notifiable disease reporting where feasible, death certificate data, or surveys) to monitor trends in disease incidence.
1995	Varicella vaccine is licensed for use in the United States.
1996	1-dose varicella vaccine is recommended for routine childhood vaccination in the United States.
1997	CSTE recommends that states and territories investigate all varicella-related deaths to monitor changes in varicella-related mortality and to understand why deaths occurred.
1998	CSTE recommends that states establish some form of ongoing systematic morbidity surveillance that might include aggregate case reporting, hospital discharge data review, sentinel systems, or surveys.
1999	Varicella deaths become nationally notifiable, effective January 1, 1999.
2002	CSTE recommends including varicella in the National Notifiable Diseases Surveillance System by 2003 and establishing case-based surveillance in all states by 2005.
2006	Varicella vaccination recommendation is updated to include a routine 2-dose childhood vaccination schedule in the United States.

* **Source:** adapted from CDC. Varicella surveillance practices—United States, 2004. MMWR 2006;55:1126–9.

† During 1972–1997, a total of 14 states maintained continuous varicella reporting to CDC.

**TABLE 2:** Varicella surveillance practices as reported by the 44 jurisdictions where varicella was a reportable condition — United States, 2012

	Jurisdictions reporting	
	
Type of surveillance*	No.	(%)
Statewide case-based	37	(84.1)
Regional sentinel site case-based	3	(6.8)
Outbreak	20	(45.4)
Aggregate	3	(6.8)
Other†	4	(9.1)

* Responses could include multiple types of varicella surveillance.

† Includes passive surveillance and surveillance limited to varicella deaths, hospitalizations, and outbreaks.

**TABLE 3:** Information collected by 38 jurisdictions conducting statewide or sentinel site varicella case-based surveillance — United States, 2012

Variables collected by jurisdictions conducting varicella case-based surveillance*	Jurisdictions reporting	

	No.	(%)
**Demographic information**		
Age	37	(97.4)
Sex	37	(97.4)
Race/Ethnicity	37	(97.4)
Country of birth	25	(65.8)
**Clinical information**		
Rash onset date	36	(94.7)
Disease severity	32	(84.2)
Location of rash (generalized, localized)	24	(63.2)
Types of lesions (macules, papules, vesicles)	24	(63.2)
Fever	28	(73.7)
Complications	27	(71.0)
Immunocompromised	24	(63.2)
Treatment (medication, type)	22	(57.9)
Pregnancy status	28	(73.7)
Past history of varicella disease	30	(79.0)
Laboratory testing for varicella performed	36	(94.7)
**Varicella vaccination history**		
Received varicella vaccine	36	(94.7)
No. of doses received and dates	35	(92.1)
**Epidemiologic data**		
Epidemiologic link	30	(79.0)
Transmission setting	27	(71.0)
Outbreak association	33	(86.8)
**Outcome**		
Hospitalized	35	(92.1)
Died	34	(89.5)

* Respondents were able to select more than one variable.

## Footnotes

* CDC has developed a document to provide guidance for investigating and managing varicella outbreaks, available at http://www.cdc.gov/chickenpox/outbreaks/downloads/manual.pdf.

† A varicella HL7 message mapping guide is available at http://www.cdc.gov/phin/library/guides/varicella_message_mapping_guide_v2_01.pdf.
